# Aortic stiffness as an independent predictor of cardiac function and cerebral white matter hyperintensities in diabetes mellitus assessed by Magnetic Resonance Imaging

**DOI:** 10.1186/1532-429X-11-S1-P169

**Published:** 2009-01-28

**Authors:** Saskia GC van Elderen, Anne Brandts, Jos Westenberg, Jeroen van de Grond, Mark van Buchem, Jan Smit, Albert de Roos

**Affiliations:** Leids Universitair Medisch Centrum, Leiden, Netherlands

**Keywords:** Left Ventricular Ejection Fraction, Aortic Arch, Arterial Stiffness, Pulse Wave Velocity, Left Ventricular Mass

## Introduction

Diabetes Mellitus (DM) causes damage in multiple organs and the vessels due to the metabolic state of hyperglycemia and insulin resistance. Increased arterial stiffness may be an important pathway linking DM to the increased cardiovascular (CV) risk by mechanisms of increased cardiac afterload, augmentation of pulse pressure, a subsequent transmission of high pulsatile flow to the end organs and furthermore as a representative of arteriosclerosis. Magnetic Resonance (MR) assessment of aortic pulse wave velocity (PWV) offers a good reproducible non-invasive tool for the detection of arterial stiffness. Furthermore MRI is an accurate tool for the detection of cardiac dysfunction and vascular cerebral lesions, like white matter hyperintensities (WMHs). To our knowledge, MRI has not previously been used to simultaneously determine end organ damage in the aorta, heart and the brain in patients with DM. We hypothesize that there is an important role for DM induced aortic stiffness in the underlying mechanism of cardiac failure and cerebral white matter hyperintensities in DM patients.

## Purpose

Accordingly, the purpose of this study is to examine the possible association between aortic PWV, cardiac left ventricular (LV) function and cerebral white matter hyperintensities in DM patients using MRI.

## Methods

MRI of the aorta, the heart and the brain was performed in 81 consecutively included subjects with proven DM (50 men; mean age 48 ± 12 years). PWV (defined as the propagation speed of the systolic wave front) was assessed using velocity-encoded MRI at two predefined levels in the aorta: in the aortic arch and the descending aorta. Short-axis MRI using a steady-state free precession sequence was performed to assess LV ejection fraction and LV mass. Transmitral flow measurements were performed by means of velocity-encoded MRI for the evaluation of LV diastolic function. WMHs on spin-echo T2-weighted and FLAIR sequences were separately evaluated according to their two anatomical subtypes – periventricular (pv) and subcortical (sc) – and respectively quantified using the Fazekas classification. Spearman correlation, independent student t-test, multivariate linear and logistic regression models were applied for statistical analysis.

## Results

PWV in the aortic arch was significantly associated with LV diastolic function (r = -0.619, p = 0.000), LV ejection fraction (r = -0.227, p = 0.042) and periventricular WMHs (p = 0.006). PWV in the descending aorta was significantly associated with LV diastolic function (r = -0.567, p = 0.000) and subcortical WMHs (p = 0.002). Furthermore, in multivariate regression analysis with confounding age, gender, smoking and duration of DM, PWV in the aortic arch was an independent predictor of LV diastolic function (R = 0.803, p = 0.002), LV ejection fraction (R = 0.471, p = 0.001), LV mass indexed for body surface area (R = 0.707, p = 0.004) and pv WMHs (OR = 1.329, p = 0.023).

## Conclusion

Stiffening of the aorta is associated with cardiac LV dysfunction and lesions of the cerebral white matter in DM. Moreover, PWV of the aortic arch is an independent predictor of cardiac LV diastolic and systolic function, LV mass and cerebral periventricular white matter hyperintensities in DM patients.

